# mGluR5 binding changes during a mismatch negativity task in a multimodal protocol with [^11^C]ABP688 PET/MR-EEG

**DOI:** 10.1038/s41398-021-01763-3

**Published:** 2022-01-10

**Authors:** Cláudia Régio Brambilla, Tanja Veselinović, Ravichandran Rajkumar, Jörg Mauler, Andreas Matusch, Andrej Ruch, Linda Orth, Shukti Ramkiran, Hasan Sbaihat, Nicolas Kaulen, Nibal Yahya Khudeish, Christine Wyss, Karsten Heekeren, Wolfram Kawohl, Elena Rota Kops, Lutz Tellmann, Jürgen Scheins, Frank Boers, Bernd Neumaier, Johannes Ermert, Markus Lang, Stefan Stüsgen, Hans Herzog, Karl-Josef Langen, N. Jon Shah, Christoph W. Lerche, Irene Neuner

**Affiliations:** 1 Institute of Neuroscience and Medicine, INM-4, Forschungszentrum Jülich GmbH, Jülich, Germany; 2 Department of Psychiatry, Psychotherapy and Psychosomatics, RWTH Aachen University, Aachen, Germany; 3 JARA – BRAIN – Translational Medicine, Aachen, Germany; 4 Institute of Neuroscience and Medicine, INM-2, Forschungszentrum Jülich GmbH, Jülich, Germany; 5 Department of Psychiatry, Psychotherapy and Psychosomatics, University Hospital of Psychiatry, Zurich, Switzerland; 6 Department of Psychiatry and Psychotherapy I, LVR-Hospital, Cologne, Germany; 7 Clienia Schlössli AG, Oetwil am See, Zurich, Switzerland; 8 Institute of Neuroscience and Medicine, INM-5, Forschungszentrum Jülich GmbH, Jülich, Germany; 9 Department of Nuclear Medicine, RWTH Aachen University, Aachen, Germany; 10 Institute of Neuroscience and Medicine, INM-11, Forschungszentrum Jülich GmbH, Jülich, Germany; 11 Department of Neurology, RWTH Aachen University, Aachen, Germany; 12 Present Address: Department of Psychiatry, Psychotherapy and Psychosomatics, University Hospital of Psychiatry, Zurich, Switzerland

**Keywords:** Diagnostic markers, Molecular neuroscience

## Abstract

Currently, the metabotropic glutamate receptor 5 (mGluR5) is the subject of several lines of research in the context of neurology and is of high interest as a target for positron-emission tomography (PET). Here, we assessed the feasibility of using [^11^C]ABP688, a specific antagonist radiotracer for an allosteric site on the mGluR5, to evaluate changes in glutamatergic neurotransmission through a mismatch-negativity (MMN) task as a part of a simultaneous and synchronized multimodal PET/MR-EEG study. We analyzed the effect of MMN by comparing the changes in nondisplaceable binding potential (BP_ND_) prior to (baseline) and during the task in 17 healthy subjects by applying a bolus/infusion protocol. Anatomical and functional regions were analyzed. A small change in BP_ND_ was observed in anatomical regions (posterior cingulate cortex and thalamus) and in a functional network (precuneus) after the start of the task. The effect size was quantified using Kendall’s W value and was 0.3. The motor cortex was used as a control region for the task and did not show any significant BP_ND_ changes. There was a significant ΔBP_ND_ between acquisition conditions. On average, the reductions in binding across the regions were - 8.6 ± 3.2% in anatomical and - 6.4 ± 0.5% in the functional network (*p* ≤ 0.001). Correlations between ΔBP_ND_ and EEG latency for both anatomical (*p* = 0.008) and functional (*p* = 0.022) regions were found. Exploratory analyses suggest that the MMN task played a role in the glutamatergic neurotransmission, and mGluR5 may be indirectly modulated by these changes.

## Introduction

Glutamate is generally acknowledged as the most important excitatory neurotransmitter for normal brain function. Nearly all excitatory neurons and over half of all brain synapses in the central nervous system are glutamatergic [1]. Furthermore, an increasing number of studies have confirmed abnormal glutamatergic neurotransmission in several mental disorders such as schizophrenia, depression, mood disorders, sleep deprivation, and addiction [2–6]. As a result, interventions aimed at targeting the glutamate system are currently under development [7]. Prior to 2006, it was not possible to measure fluctuations in endogenous glutamate in vivo due to the lack of radiotracers for assessing the sensitivity of glutamate receptors to changed glutamate levels induced by a drug or stimuli tasks [8]. However, more recently, the use of 3-(6-methyl-pyridin-2-ylethynyl)-cyclohex-2-enone-O-[^11^C] methyloxime, [^11^C]ABP688 [9], and similar radiotracers have proved to be highly selective antagonistic PET agents for mGluR5. DeLorenzo and colleagues found a significant reduction in the nondisplaceable binding potential (BP_ND_) of [^11^C]ABP688 in ten healthy nonsmokers after the administration of ketamine [10]. Based on their findings, they hypothesized that the displacement of [^11^C]ABP688 may occur as a result of indirect competition and/or receptor internalization.

Similarly, Esterlis and colleagues reported a significant reduction in mGluR5 availability in patients with a major depressive disorder and healthy controls following the administration of ketamine [11]. Furthermore, the superiority of [^11^C]ABP688 in drug-challenge paradigms designed to probe glutamate transmission when compared with [^18^F]FPEB was proven [12]. Thus, [^11^C]ABP688 has emerged as a useful radiotracer and has expanded the possibilities for in-depth research into the role of glutamatergic neurotransmission in both psychiatric disorders and healthy processes considerably.

One of the most commonly used and informative biological indicators of glutamatergic neurotransmission is the mismatch-negativity (MMN) paradigm [13]. MMN is an event-related potential (ERP) component, elicited by violations of a standard, that reflects the brain’s ability to perform comparisons between repetitive stimuli, thereby providing an electrophysiological index linked to sensory learning and perception [14, 15]. This electrophysiological response can be detected using electroencephalography (EEG) or magnetoencephalography (MEG). The MMN is elicited by sudden changes in stimulation and shows the strongest intensity in temporo-frontal areas of the EEG maps [16]. Studies examining MMN have been performed in many clinical applications, especially in schizophrenia, and for better comprehension of auditory perception and sensory memory representations [17–19]. Thereby, most of the studies have found significant reductions in the MMN amplitude and longer latency times in patients with schizophrenia compared with healthy volunteers. Moreover, there is robust evidence indicating that this is mainly due to a dysfunction in N-methyl-D-aspartate (NMDA) receptors and is linked to impaired cognitive performance [19–21]. Further, significant reductions in the MMN amplitude were also observed following the administration of ketamine, which acts as an antagonist of NMDA [22–25]. As mGluR5 and NMDA are functionally correlated, measuring the availability of mGluR5 may also provide valuable information about NMDA receptors.

Given the close correlation between MMN and glutamatergic neurotransmission, a better understanding of the dynamics in this interaction is of high scientific interest. However, until now, these kinds of investigations were hardly possible due to technological limitations. The development of the simultaneous trimodal neuroimaging approach with positron-emission tomography (PET), magnetic resonance imaging (MR), and EEG (PET/MR-EEG) has accelerated research in this field in recent years. The main advantage of this approach is that structural and functional (via fMRI) and metabolic (via PET) data can be acquired simultaneously under the same physiological and psychological conditions [26].

In this work, we investigated the feasibility of simultaneous PET/MRI–EEG acquisition using [^11^C]ABP688 to assess changes in glutamatergic neurotransmission through an MMN task. In order to determine any MMN effects in subjects during the PET measurement, we compared the changes in the BP_ND_ of [^11^C]ABP688 between the pretask resting-state moments and MMN-task moments.

## Materials and methods

### Radiochemistry

Radiosynthesis of [^11^C]ABP688 was performed according to [27]. The average molar activity at the injection time was 101.70 ± 45.33 MBq/nmol.

### Subjects

Seventeen healthy, male, nonsmoker [8], and smoker [9] volunteers with a mean age of 38.47 ± 11.38 years were scanned in a single, multimodal session using a 3 T hybrid MR-BrainPET insert system (Siemens, Germany) [28] equipped with a 64-channel MR-compatible EEG system (Brain Products, Germany). The study was approved by the Ethics Committee of the Medical Faculty of the RWTH Aachen University and the German Federal Office for Radiation Protection. All subjects were scanned once, with an injected activity not exceeding 600 MBq. The MINI International Neuropsychiatric Interview was used to confirm that none of the subjects had a history of psychiatric disorders. Verbal and written informed consent were obtained from each volunteer according to the Declaration of Helsinki.

### Multimodal acquisition

#### PET and bolus-infusion protocol

Our protocol was optimized and updated based on a previous publication [29]. Two syringes containing the radiotracer solution for the bolus and for the infusion were prepared 10 min prior to the bolus injection. The bolus injection (50% of total activity), followed by 65 min of infusion (activity in 100 ml of NaCl^1^; infusion pump at 92 ml/h rate), was administered after positioning the subject in the scanner. The average injected activity per subject was 468.50 ± 66.03 MBq. A distribution equilibrium was observed 30 min after the bolus injection [30, 31]. Starting simultaneously with the bolus injection, the PET data were acquired in list mode for 65 min.

Venous blood samples were taken at 2, 5, 10, 15, 20, 25, 30, 35, 40, 45, 50, 55, and 60 min after the bolus injection. Blood samples were centrifuged, and the plasma-activity concentration was measured in a gamma counter (Wallac 1480 Wizard). Furthermore, for the correction of tracer metabolization, the parent compound was separated from the metabolites in each sample by a solid-phase extraction using cartridges (Waters Sep-Pak^®^ tC18).

#### MMN paradigm

The MMN paradigm consisted of changes in tone duration (standard = 50 ms, deviant = 100 ms). Auditory stimuli (1 kHz, 10 ms attack/decay) were presented in alternating sequences of mostly 50 ms with fewer 100 ms in positions 9 up to 16 after the standard tone (stimulus-onset asynchrony of 0.85 ± 0.05 s). The deviant positions were pseudo-randomized (only two equal positions following each other). A total of 1410 trials (8% deviant, 92% standard) were presented [32]. The subjects were instructed not to pay attention to the tones, and a silent video was presented to them for distraction.

#### EEG

Signals were recorded simultaneously with fMRI and PET during the MMN-task paradigm using a 64-channel MR-compatible EEG system. The EEG cap (BrainCap MR, EasyCap) consisted of 63 scalp electrodes, positioned according to the 10% system, covering the 10/20 area. One additional electrode was used for recording the electrocardiogram (ECG). Prior to recording, electrolyte gel (ABRALYT 2000, EASYCAP) was applied to each subject’s head to increase the conductivity between scalp and EEG electrodes, followed by the placement of the EEG cap. The impedance of all recording electrodes was below 10 kΩ. The EEG data were recorded with a Brain Vision Recorder (Brain Products). The sampling rate of the EEG recording was 5 kHz. In order to avoid vibration effects, the helium pump of the MRI system was switched off during EEG recording.

#### MRI

Anatomical images were acquired with the magnetization-prepared rapid gradient-echo (MPRAGE) sequence (TR = 2250 ms, TE = 3.03 ms, 176 sagittal slices, 1 mm slice thickness, GRAPPA factor 2) after the tracer bolus injection. To provide functional images, a T_2_^*^-weighted echo-planar imaging (EPI) sequence (TR = 2.2 s, TE = 30 ms, FOV = 200 mm, slice thickness of 3 mm) was acquired when the tracer reached equilibrium. Single-voxel spectra were measured using a standard point-resolved spectroscopy (PRESS) sequence (after MPRAGE and before EPI acquisitions).

**Fig. 1:**
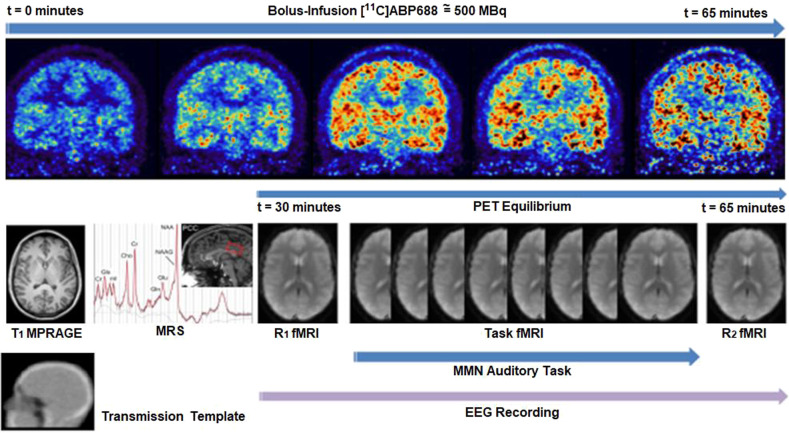
Design of a measurement session. Schematic representation of the multimodal imaging approach.

The EEG, MMN paradigm, and the fMRI were all synchronized to the PET acquisition. The first resting state (RS_1_) started 30 min after the bolus injection (tracer equilibrium), followed by the MMN task and the second resting-state acquisition (RS_2_). An MPRAGE image was used as an anatomical reference and for PET-attenuation correction [33]. Figure 1 shows the multimodal protocol in a schematic way.

### PET image reconstruction and processing

**Fig. 2:**
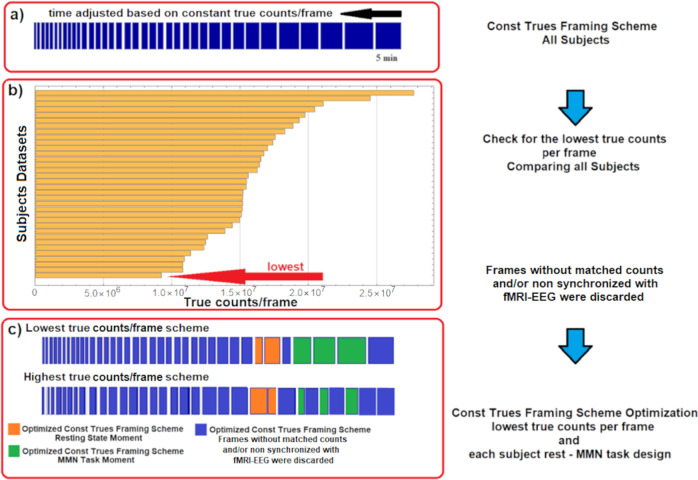
PET-matched framing scheme applied in this [^11^C]ABP688 study. **a** General frame scheme with variable time-frame lengths, enabling constant true counts/frame. **b** Comparison of all subjects to find the lowest-average true counts per frame. **c** Two individual Const Trues framing schemes showing the optimization to match the lowest-average true counts per frame between subjects and synchronized with the different acquisition moments: pretask resting state and the MMN task.

As shown in Fig. 2(a), the image reconstruction was performed using 3D-OP-OSEM (2 subsets, 32 iterations), with an isotropic voxel of 1.25 mm, 153 slices, 256 × 256 voxels. A framing scheme was used to reduce reconstruction bias and the BP_ND_ errors were kept constant in the relevant acquisition interval [34]. Based on this scheme, PET true counts per frame based on this scheme were matched between subjects and synchronized with the different acquisition moments: pretask resting state and the MMN-task according to Fig. 2(c). For the subject with the lowest average of true counts, a total of 9.35 × 10^6^ counts were registered in the last 5 min of the acquisition (see Fig. 2(b)), which corresponded with the third MMN-task interval. This value was taken as the fixed reference count per frame, and the three task-interval time fractions from the subject where this low limit occurred were computed to give 0.267, 0.324, and 0.408 for the entire task interval. The task intervals were computed for each subject according to the total length of the MMN-task interval and these fractions. The three frames within the task time interval were then grown symmetrically around the center of these three intervals until the reference count (9.35 × 10^6^) was reached, see Fig. 2(c). Frames without matched counts and/or not synchronized with fMRI-EEG were discarded. In this way, all seventeen subjects had the same number of counts during the RS_1_ moments, and the reconstruction bias was equal for all [34]. In addition, the three frames representing the MMN-task moments were aligned at the same time during the task for all subjects. The images were corrected for attenuation, random and scattered coincidences, and dead time. Post-processing with a 2.5 mm 3D Gaussian filter was applied. In addition, head motion correction based on the multiple-acquisition-frame (MAF) reconstruction scheme [35] was performed [36, 37].

### Metabolite correction and plasma analysis

Aliquots of 400 μL plasma were diluted to 2.7 mL in water, loaded onto an injection loop, and passed through the SPE cartridge. This was followed by 5 mL of water using a motor syringe (7.7 mL over 2 min). Both the 2 ml aliquot of the eluate (totaling 7.7 mL and containing the metabolites) and the cartridge (containing the parent compound) were measured using a gamma borehole counter (ISOMED 2400) for a period of 60 s each. All activity concentrations were decay corrected according to the bolus-injection time. Biexponential curves were fitted to the fraction of the parent compound and to the total plasma-activity concentration. The corrected plasma activity curve was computed as the product of the total fitted plasma and the fitted fraction of the parent compound in the extract.

### Equilibrium quality analysis

The [^11^C]ABP688 parent compound time-activity curves (TACs) were normalized to their average values from 30 to 60 min, and the corresponding normalized radioactivity ranges were evaluated according to Eq. 1.

1 $${{{\mathrm{Normalized}}}}\;{{{\mathrm{Range}}}}\left[ {{{\mathrm{\% }}}} \right]\left[ {\frac{{\left( {{{{\mathrm{max}}}} - {{{\mathrm{min}}}}} \right)}}{{\left( {{{{\mathrm{max}}}} + {{{\mathrm{min}}}}} \right)}}} \right] \times 100.$$

The distribution volume (V_T_) of [^11^C]ABP688 was calculated (Eq. 2) for two anatomical reference and control regions (cerebellum gray matter and motor cortex) where both the tissue TAC and the metabolite-corrected plasma curve were flat. This indicates an equilibrium at the respective time points.

2 $${{{\mathrm{V}}}}_{{{\mathrm{T}}}}[{{{\mathrm{ml}}}}/{{{\mathrm{cm}}}}^3] = \frac{{{{{\mathrm{C}}}}_{{{\mathrm{T}}}}}}{{{{{\mathrm{C}}}}_{{{\mathrm{P}}}}}}$$

C_T_ denotes the [^11^C]ABP688 concentration in the brain tissue, and C_P_ represents the unmetabolized [^11^C]ABP688 parent compound in the plasma. The profiles were accepted as being flat if the normalized-range values from 30 to 60 min varied by no more than 10%. The TACs in this interval and from both regions were also adjusted using a linear regression in the average group curve, and the slopes were evaluated with reference to the equilibrium stability. Both regions were required to have a slope that was not significantly different from zero when a 95% confidence interval in the linear-regression analysis was considered.

### PET quantitative data analysis

PNEURO/PMOD software v.3.9 was used to define the volumes of interest (VOIs) with T1 MPRAGE images serving as an anatomical reference. All images were processed in the PET subject’s space, and the Hammers atlas [38] was applied for activity-concentration analysis. All VOIs in the gray matter cortex (GM) were defined with the maximum-probability operation. Furthermore, functional masks [39] were applied following conversion to the PET space. Finally, anatomical VOIs, comprising the whole brain, frontal left, frontal right, medial orbitofrontal cortex (mOFC), parietal left, parietal right, temporal left, temporal right, medial temporal, primary auditory, anterior cingulate cortex (ACC), posterior cingulate cortex (PCC), caudate, putamen, thalamus, motor cortex, and cerebellum GM, as well as functional VOIs, comprising the default-mode network (DMN), auditory network (AN), primary visual network (pVN), high visual network (hVN), visuospatial network (VN), language network (LN), salience network (SN), basal ganglia network (BgN), precuneus network (PN), and left and right executive control networks (LECN and RECN), were applied to extract the corresponding activity concentrations. To extract the activity concentrations from GM regions, only voxels with more than 50% probability of belonging to GM were considered. In previous studies, the cerebellum GM has been chosen as the most appropriate reference region for human-brain studies on mGluR5 [2, 3, 40, 41]. In all cases, the nondisplaceable binding potential (BP_ND_) of [^11^C]ABP688 was estimated as follows (Eq. 3):

3 $$\left[ {\left( {\frac{{C_T}}{{C_{\rm{Cerebellum}}}}} \right) - 1} \right]$$

where C_T_ is the total radioligand concentration in the region of interest and C_Cerebellum_ is the radioligand concentration in the cerebellar GM-reference tissue assumed to be nondisplaceable.

According to the aforementioned framing-scheme definition, the BP_ND_ was estimated for each of the acquisition moments: RS_1_, MMN_1_, MMN_2_, and MMN_3_. It was defined in this way to enable comparisons between RS_1_ and each of the MMN moments, thus allowing the detection of the part of the MMN interval that has a significant effect when compared with the RS_1_ baseline. The motor cortex was taken as a task-reference region for the analysis because, due to the nature of the MMN elicitation, it is not expected to be activated or show tracer displacements during the MMN task.

### EEG processing

The EEG data were processed using the MATLAB-based (v.9.2, R2017a) software packages EEGLAB^2^ v.13 [42]. The recorded raw EEG data were imported to EEGLAB and downsampled to 1024 Hz. The gradient artifacts that were mixed with the EEG signals linearly due to the switching of the magnetic gradients were removed using the FASTR tool [43]. The EEG data were again downsampled to 256 Hz and filtered between 1 and 20 Hz using a Hamming windowed sinc finite impulse-response filter. Bad channels were identified and removed using the EEGLAB function clean_rawdata. Artifact subspace reconstruction [44] was performed to remove the nonstationary artifacts, such as head movement. An adaptive optimal basis-set algorithm [45] was used to remove the ballistocardiogram artifacts in the EEG data caused by pulsatile blood flow. Ocular artifacts were removed by using an automatic blind-source separation algorithm [46]. EEG signals were re-referenced to an average reference. In order to remove further residual artifacts, independent component analysis [47] was performed, and the multiple artifact-rejection algorithm [48] was used to classify and remove artifactual components. The EEG signals were segmented between 200 ms prior to—and 400 ms after the standard and frequent auditory stimuli marker position. Baseline correction was performed using 100 ms data prior to the stimuli-marker. The segmented EEG data were averaged over trials. MMN latency was measured between 100 ms and 300 ms post deviant tone at the C_Z_, P_Z_, and F_Z_ EEG channels individually and at the average of the F_3_, F_Z_, F_4_, C_3_, C_Z_, and C_4_ channels.

### Statistical analysis

Statistical analysis was performed using the Statistical Package v.25 (IBM SPSS Inc., Chicago, IL). Differences between acquisition conditions were compared using the nonparametric Wilcoxon Rank test (between baseline and each task moment separately). Additionally, baseline and task moments were evaluated in repeated measures using the Friedman test. Within-subject SE-bar standardization was performed according to [49]. Corrections for multiple comparisons using the Bonferroni method were applied in the evaluation for task effects in the within-subject design, and the updated significance was 0.001. The Kendall’s W coefficient was used to evaluate the effect size. It tests the ΔBP_ND_ in each region and gives a value that ranges between 0 and 1. A Kendall’s W interpretation of 0.2 represents a small effect, 0.5 a moderate effect, and above 0.8 a strong effect. The extent of BP_ND_ changes during the MMN (calculated as $\left[ {\left( {{\rm{BP}}_{{\rm{ND}}_{\rm{MMN}}} - {\rm{BP}}_{{\rm{ND}}_{\rm{Baseline}}}} \right){{{\mathrm{/}}}}{\rm{BP}}_{{\rm{ND}}_{\rm{Baseline}}}} \right] \ast \left. {100} \right)$ in regions that showed significant binding changes) was examined for an existence of a significant Pearson’s correlation with the EEG-latency times during the MMN task. Due to the purely exploratory approach, these correlations were not corrected for multiple testing.

This study describes the effect of the MMN paradigm on the BP_ND_, and for regions that may show significant task effects, it also presents correlations between the binding changes and the EEG-latency times represented by the time range from the onset of stimuli until the MMN auditory stimulation peak.

## Results

### Equilibrium Quality

**Fig. 3:**
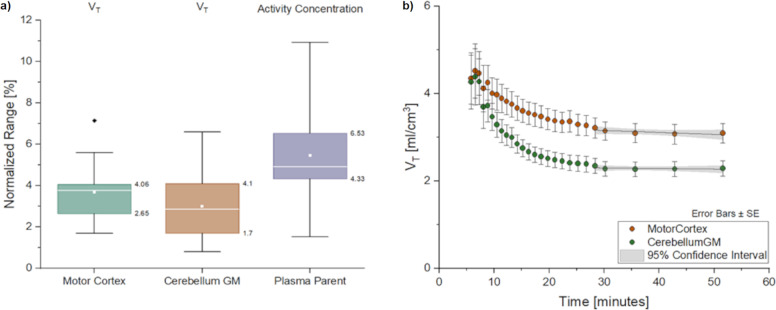
Equilibrium quality evaluation/inspection represented by regional cerebral uptake of [C]ABP688 expressed as distribution volume referenced to plasma parent, V. **a** Normalized-range variations of the V_T_ values in the plateau range (30–60 min) for the motor cortex and cerebellum GM-control regions during the equilibrium phase. The plasma parent compound normalized activity-concentration range for evaluating the equilibrium quality is also shown. **b** Average V_T_ curves and the linear regression applied to the data plateau range (30–60 min) for the slope analysis. SE = standard error of the mean (mean/$\sqrt n$), where *n* = sample size.

Figure 3a shows the percentual normalized range values of the V_T_ for the two anatomical reference and control regions, the cerebellum GM and the motor cortex, and the blood plasma-activity concentration for all subjects. For all quantities measured, values were lower than 5%, except for variations of 5.46 ± 2.38% in the plasma values during the equilibrium phase.

Figure 3b shows the average V_T_ vs. time curves for both the reference and task-control regions and the adjusted linear-regression line in the expected equilibrium interval. Both regions showed slope values that were not significantly different from zero based on a 5% significance level in the analysis.

Slope values in both the reference and control regions are given in Table 1. It can be noted that no significant differences in the slopes were found for either region. This means that the equilibrium was well established, and these regions can serve as reference and task-control regions.

**Table 1:** Slope values and SE in control regions.

Regions	Value	Standard error	t-Value	Prob > $\left| {\frac{{\it{\upalpha }}}{2}} \right|$
motor cortex	−0.004	0.002	−1.962	0.144
cerebellum GM	−0.001	0.001	−0.751	0.506

### Binding changes ΔBP_ND_ during the MMN task

The task effects were analyzed separately for anatomical and functional VOIs by estimating the BP_ND_ using the simple ratio method. The statistical comparisons were performed using nonparametric Wilcoxon and Friedman tests with *p* ≤ 0.001 (16 anatomical regions and 3 comparison pairs) and *p* ≤ 0.002 (11 functional regions and 3 comparison pairs) after Bonferroni correction. Kendall’s W coefficient was used to measure the effect size for each region. Table 2 shows the results for anatomical regions, and Table 3 shows the results for functional regions. Note that the motor cortex is the control region for the task-effect analysis and should not show any effect.

**Table 2:** Task effect results for anatomical brain regions analyzed with the Wilcoxon and Friedman tests and corrected using the Bonferroni method.

Brain region	*p* value – Wilcoxon RS_1_ x MMN_1_/MMN_2_/MMN_3_	*p* value – Friedman RS_1_ x MMN_1_/MMN_2_/MMN_3_	Effect’s size Kendall’s W	Acquisition moment occurrence
Whole brain GM	0.652/0.266/0.027	0.678	0.030	–
Frontal left	0.246/0.021/0.003	0.004	0.258	–
Frontal right	0.209/0.024/0.002	0.002	0.283	–
mOFC	0.435/0.619/0.102	0.630	0.034	–
Parietal left	0.148/0.027/0.005	0.007	0.236	–
Parietal right	0.209/0.075/0.006	0.083	0.131	–
Temporal left	0.356/0.193/0.055	0.487	0.048	–
Temporal right	0.462/0.136/0.013	0.208	0.089	–
Temporal Med	0.981/0.758/0.093	0.615	0.035	–
Primary auditory	0.553/0.209/0.061	0.678	0.030	–
ACC	0.162/0.013/0.011	0.010	0.222	–
PCC	0.035/0.024/0.001*	0.005	0.255*	MMN_3_
Caudate	0.266/0.113/0.006	0.039	0.164	–
Putamen	0.522/0.286/0.227	0.796	0.020	–
Thalamus	0.017/0.227/0.001*	0.001*	0.347*	MMN_3_
Motor cortex	0.227/0.245/0.193	0.615	0.035	–

Effect size was evaluated using Kendall’s W.

*Significant effects (p ≤ 0.001).

**Table 3:** Task effect results for functional brain regions analyzed with the Wilcoxon and Friedman tests and corrected using the Bonferroni method. Effect size was evaluated using Kendall’s W.

Brain region	*p* value – Wilcoxon RS_1_ x MMN_1_/MMN_2_/MMN_3_	*p* value – Friedman RS_1_ x MMN_1_/MMN_2_/MMN_3_	Effect’s size Kendall’s W	Acquisition moment occurrence
DMN	0.162/0.027/0.005	0.029	0.203	–
AN	0.148/0.981/0.084	0.467	0.062	–
pVN	0.868/0.102/0.009	0.225	0.197	–
hVN	0.492/0.523/0.005	0.808	0.147	–
VN	0.061/0.044/0.004	0.090	0.153	–
LN	0.331/0.162/0.011	0.090	0.114	–
SN	0.522/0.055/0.006	0.090	0.181	–
BgN	0.831/0.687/0.019	0.225	0.106	–
PN	0.068/0.004/0.001*	0.0005*	0.307*	MMN_3_
LECN	0.245/0.049/0.004	0.029	0.175	–
RECN	0.943/0.463/0.013	0.225	0.178	–
Motor cortex	0.209/0.246/0.193	0.467	0.042	–

*Significant effects (*p* ≤ 0.001).

The effect size due to the MMN task is classified as a small effect according to the Kendall’s W coefficient scale. This is also true when compared with the effect sizes caused by pharmacological challenges obtained, e.g., with ketamine and [^11^C]ABP688. These challenges showed effects of 0.88, as represented by a similar scale [50]. The ketamine effects are considered high and are in agreement with the high surge of glutamate release in the brain, as identified in a PET-depression study [11].

**Fig. 4:**
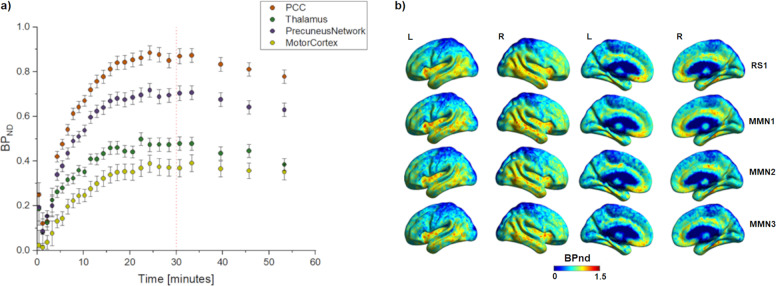
Task effect results shown by the regional and voxel-wise cerebral uptake of [C]ABP688 expressed as distribution volume referenced to cerebellum grey matter, BP. **a** BP_ND_ curves for the anatomical and functional brain regions analyzed. Significant effects can be seen in the healthy group. The red line marks the start point of tracer equilibrium phase. After 30 min, the first two time points represent RS_1_ and the remaining three time points represent the MMN task moments (MMN_1_, MMN_2_, and MMN_3_). **b** BP_ND_ parametric images for the group average per acquisition moment.

Figure 4a shows the BP_ND_ curves for the anatomical and functional regions with significant differences in binding during the MMN task when compared with the motor cortex. Figure 4b shows parametric images (sagittal plane with lateral and medial) of the average BP_ND_ for the different acquisition moments.

It is possible to identify a slight reduction in the BP_ND_ values during the transition between MMN_2_ and MMN_3_. These changes are hardly observable with the naked eye. However, they can be noted by comparing the BP_ND_ curves (Fig. 4a), proving that the values decrease (−6.39 ± 2.49% PCC, 3−10.87 ± 6.34% thalamus and −6.7 ± 3.10% PN) slightly over time. Also, the Kendall’s W scale showed that the MMN task had a low effect over time, which explains the very tiny change between the acquisition moments.

### Correlation between ΔBP_ND_ and EEG-latency times during the MMN task

**Fig. 5:**
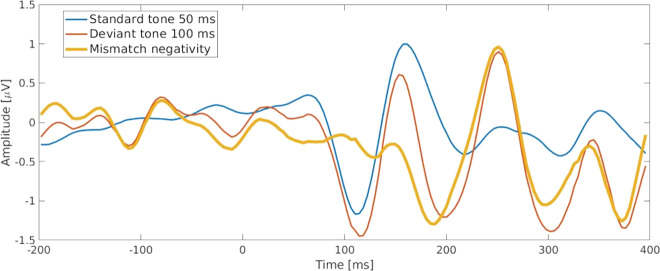
Auditory-evoked potential (AEP) of standard and deviant tones during the MMN paradigm calculated by averaging F_3_, F_Z_, F_4_, C_3_, C_Z_, and C_4_ channels. Additionally, the mismatch-negativity difference wave obtained by subtracting the standard from deviant event-related potentials is also shown.

For the three regions that showed binding changes, the PCC and PN showed correlations with the EEG P_z_ electrode-latency times of *r* = − 0.549; *p* = 0.023, and *r* = − 0.552; *p* = 0.022, with a significance of *p* < 0.05. In the thalamus, the correlation between the extent of the BP_ND_ change during the MMN and the average of latencies for three central (C_3_, C_4_, and C_z_) and three frontal (F_3_, F_4_ and F_z_) electrodes was *r* = 0.623, *p* = 0.008, with a significance of *p* < 0.01. Figure 5 shows the auditory-evoked potential during the task as an average of the EEG channels and the MMN wave.

## Discussion

This in vivo imaging study used simultaneous PET/MR-EEG imaging in a group of healthy male subjects with the aim of revealing the first evidence for MMN task-induced changes in glutamatergic neurotransmission. After corrections for multiple comparisons using the Bonferroni method, we observed a significant decrease in the availability of mGluR5 in the thalamus, the PCC (except in Friedman analysis), and the PN during the MMN task. The effect size of the observed changes was small (W = 0.3) when compared with effect sizes as a result of pharmacological challenges with ketamine [10, 11], where effects of 0.88 could be demonstrated [50]. The authors attribute the decrease in mGluR5 availability to the rapid surge in extracellular glutamate leading to mGluR5 downregulation or internalization. The glutamate-surge hypothesis originates from rodent data showing that a single administration of ketamine rapidly induces increases in glutamate efflux [51] and cycling [52]. Accordingly, the change in mGluR5 binding observed in our study during the MMN task could be related to an increase in glutamate in the regions pointed out, although to a much smaller extent.

Separate temporal and frontal generators of MMN have been consistently identified using various source-localization methods [53]. A temporal generator for MMN is localized in the primary auditory cortex and is considered to be the primary generator responsible for MMN elicitation [54]. However, a recent study with nonhuman primates showed that the thalamic region is also involved in MMN [55]. The involvement of the auditory thalamus was previously found in healthy human participants [56], and numerous investigations suggest that the human thalamus serves as a global hub that is connected with the entire cortex, relaying sensory information to the cortex and mediating the transmission of cortico-cortical information [57]. Moreover, the thalamus could be indicated as being a critical integrative hub for functional brain networks engaged with multiple cognitive functions [58]. The auditory thalamus (medial geniculate body) represents the primary sensory input to auditory-cortex processing [59]. Thus, it is the information bottleneck for neural representations of sounds being sent to the auditory cortex and plays a critical role in the complex auditory processing [59], which forms an important part of the MMN. Correspondingly, our findings confirm thalamic involvement in the MMN and further relate this involvement to glutamatergic neurotransmission.

We observed a significant reduction in the binding potential for the PCC and PN, which is in accordance with previously reported involvement of these regions in the mismatch response [60].

The PCC is a highly anatomically connected part of the posteromedial cortex and represents a central part of the DMN [61]. Moreover, some evidence suggests that the PCC plays a more direct role in regulating the focus of attention [62]. Thus, the finding of a significant decrease in BP_ND_ in the PCC during the MMN task might be understood as an effect of a further shift in attention due to the tone alterations.

The PN is a network centered at the precuneus. It has recently been argued that it is functionally independent of the DMN, although this remains controversial due to the considerable extent of spatial adjacency and overlap [63]. However, the core region of the PN, the precuneus, is known to be widely connected with other regions in the brain [64, 65], and numerous studies have indicated its involvement in a wide range of cognitive processes [66]. Also, a strong structural and functional connectivity of the precuneus has been shown with the thalamus and the DMN [64]. Furthermore, previous investigations have demonstrated that the PN plays an important role in the detection of novelty [67]. Accordingly, the observed reduction in the binding in our study may be an expression of the glutamate surge in the PN in response to the tones heard during the MMN task.

Given that [^11^C]ABP688 and glutamate bind at different sites on the receptor, this decrease cannot be due to direct competition, and the mechanism responsible for the changes in binding after an MMN challenge relating to mGluR5 is still not completely understood. After observing a significant ketamine-induced reduction in mGluR5 availability, Esterlis et al. hypothesized that binding changes may be due to increased mGluR5 internalization reducing affinity by altering the local intracellular milieu [11]. In our study, we demonstrated, for the first time, that this alteration may occur due to an MMN challenge. Accordingly, the exposure to a certain amount of endogenous glutamate released during the MMN task may have caused an internalization of the glutamatergic receptors. This, in turn, may have indirectly induced changes in the BP_ND_. One conceivable course of events could be that the released endogenous glutamate binds to the main site of the mGluR5 receptors, promoting their internalization or simply a conformational rearrangement, and thus (minor) structural changes, which lead to a decrease in the affinity of [^11^C]ABP688 for the receptor and therefore to a displacement into the allosteric site.

To date, the glutamatergic basis of MMN has been hypothesized through indirect reasoning from other studies [68, 69], and a direct glutamatergic readout has only been used in this context in a few cases. In their spectroscopic study, Stone and colleagues showed an association between smaller frontal MMN amplitudes and lower levels of the overlapping resonance of glutamate and glutamine in the thalamus in participants at risk of psychosis [70]. However, in their study, the recording of MMN and the spectroscopy occurred at different points in time, while in our study, all data modalities were acquired simultaneously. This allows for a higher level of synchronization, and the change in the binding could be captured exactly during the MMN task.

The observation of the tiny effect of the MMN task on the mGluR5 binding was possible due to a previous study where the reconstruction bias was minimized and kept constant during the equilibrium plateau where BP_ND_ is estimated, thus enabling the detection of changes larger than the bias range of 2.56 ± 3.92% [34]. This was achieved by applying an optimized constant trues framing scheme. In addition, since within-subject SE standardization was also applied in the analysis, the chances of finding small changes also increased due to the reduction in concomitant variance [49].

Another interesting finding from our study, which was only possible due to the multimodal, simultaneous PET/MR and EEG data acquisition, was the significant correlation between the changes in mGluR5 availability due to the MMN task (namely in regions where we observed significant BP_ND_ changes) and EEG latencies captured with several electrodes.

Hereby, the most prominent association concerned the thalamus: the higher BP_ND_ change was associated with the longer average latency time captured with three central and three frontal electrodes. The thalamus has been referred to as the gateway (or hub) of nearly all sensory inputs (except for the olfactory system) to the corresponding cortical areas through direct thalamocortical circuits [71, 72]. Consequently, thalamic operations require highly complex inhibitory activity, mainly arising from the GABAergic mechanisms within the thalamic circuits [73]. As the GABA release is strongly mediated by the mGluR activation [74], the association between the higher [^11^C]ABP688 displacement and the longer latencies might be an expression of the primary inhibitory effect of the glutamatergic surge in the thalamus.

In contrast, the greater extent of BP_ND_ changes in the PCC and PN during the MMN task was associated with shorter latencies. Considering the previous findings, where longer latencies were reported in several conditions with impaired cognitive performance (chronic schizophrenia [21], Alzheimer’s disease [75], and mild cognitive impairment [76]), and the fact that the MMN can be considered an index of the context-dependent information processing at the level of the primary and secondary auditory cortices [77], our findings provide an indication for a link between higher glutamatergic activity in the PN and PCC and faster auditive information processing. As the generation of MMN is a fully automatic process and therefore independent of the subject’s attention [78, 79], an extensive glutamatergic response to the stimulus violation in the PCC and the PN might have a decisive role for fast automatic information processing.

Despite the positive results obtained, it is important to consider that the study may be limited by the relatively small sample size and the combined analysis of smokers and nonsmokers. Consequently, additional studies are needed to understand the complex effects that the cognitive tasks involved in MMN auditory stimulation may have on the glutamatergic system when imaged with PET. Finally, although many corrections have been applied to minimize the bias caused by image reconstruction, quantification, and statistical analysis, a residual bias in the results cannot be completely excluded.

With the reported approach, our study provides evidence of a reduction in glutamatergic receptor binding and indirectly shows an active alteration in glutamatergic neurotransmission during auditory information processing in healthy subjects for the first time. In light of this, further replication is required, along with investigations into whether the extent of the changes recorded is related to cognitive abilities, or in the case of patients, whether there is a correlation with clinical symptomatology. Thus, our approach may be applied to further investigate the role of glutamatergic neurotransmission in healthy subjects and in patients with various mental disorders.

## Footnotes

1 (NaCl = 0.9% sodium chloride solution).

2 (https://sccn.ucsd.edu/eeglab/index.php).

3 (PCC showed significant result in the Wilcoxon test, but not in Friedman test).
